# Seroepidemiology of *Leptospira* Exposure in General Population in Rural Durango, Mexico

**DOI:** 10.1155/2015/460578

**Published:** 2015-07-09

**Authors:** Cosme Alvarado-Esquivel, Luis Francisco Sánchez-Anguiano, Jesús Hernández-Tinoco

**Affiliations:** ^1^Biomedical Research Laboratory, Faculty of Medicine and Nutrition, Juárez University of Durango State, 34000 Durango, DGO, Mexico; ^2^Institute for Scientific Research “Dr. Roberto Rivera Damm”, Juárez University of Durango State, 34000 Durango, DGO, Mexico

## Abstract

The magnitude of *Leptospira* exposure in rural Mexico is largely unknown. We sought to determine the seroprevalence of *Leptospira* IgG antibodies in adults in rural Durango, Mexico, and to determine the sociodemographic, behavioral, and housing characteristics of the subjects associated with *Leptospira* seropositivity. We performed a cross-sectional study in 282 adults living in rural Durango, Mexico. Sera from participants were analyzed for *Leptospira* IgG antibodies using a commercially available enzyme immunoassay. Seroprevalence association with the characteristics of the subjects was analyzed by bivariate and multivariate analyses. Of the 282 rural subjects (42.91 ± 17.53 years old) studied, 44 (15.6%) had anti-*Leptospira* IgG antibodies. Seropositivity to *Leptospira* was not associated with gender, educational level, employment, socioeconomic status, contact with animals or soil, or type of floors at home. In contrast, multivariate analysis showed that *Leptospira* exposure was associated with national trips (OR = 2.09; 95% CI: 1.05–4.16; *P* = 0.03) and poor education of the head of the family (OR = 2.96; 95% CI: 1.51–5.78; *P* = 0.001). We demonstrated serological evidence of *Leptospira* exposure in adults in rural northern Mexico. The contributing factors associated with *Leptospira* exposure found in the present study may be useful for optimal planning of preventive measures against *Leptospira* infection.

## 1. Introduction

Pathogenic spirochetes of the genus* Leptospira* are the etiological agents of the zoonotic disease called leptospirosis [1]. This zoonosis occurs worldwide [1, 2], especially in countries with tropical and subtropical climates [2]. Transmission of* Leptospira* to humans occurs by exposure to contaminated river or lake water or animals [3, 4]. Wild rodents are important reservoirs of leptospirosis [5, 6]. In fact, leptospirosis is an “emerging” zoonosis due to increased contact of humans with animals [7]. Water-borne outbreaks of leptospirosis have also been reported [8]. In addition, it has been hypothesized that soil may serve as reservoir for* Leptospira* and infectious source for leptospirosis [2]. Most infections with* Leptospira* are asymptomatic; however, some infected individuals may develop a febrile illness [9], hemorrhage [10], and multiorgan failure [11]. Furthermore, leptospirosis during pregnancy may lead to liver damage in the pregnant woman and fetal distress [12].

The seroepidemiology of* Leptospira* infection in rural adults in Mexico is largely unknown. The poor sanitation in general and the contact with river and lake water in particular may favor transmission of* Leptospira* among the general population in rural communities in Mexico. People in rural Durango have a high exposure to animals and this condition may represent a risk for* Leptospira* infection. There are not currently reliable statistics about the magnitude of* Leptospira* infection in rural Mexico. Laboratory tests for diagnosis of* Leptospira* infection in rural health centers in Mexico are lacking. Therefore, the aims of the present study were to determine the seroprevalence of* Leptospira* IgG antibodies in adults in rural Durango, Mexico, and to determine the sociodemographic, behavioral, and housing characteristics of the rural subjects associated with* Leptospira* seropositivity.

## 2. Materials and Methods

### 2.1. Study Design and Rural Population

We performed a cross-sectional study using residual serum samples obtained in 2006-2007 from rural populations in Durango State, Mexico. The original work was aimed to determine the seroepidemiology of* Toxoplasma gondii* infection in rural populations in Durango, Mexico [13]. Serum samples were collected in three rural communities: San Dimas, Villa Montemorelos, and Santa Clara. Inclusion criteria for enrollment in the survey were (1) inhabitants of rural Durango, Mexico; (2) age of 18 years and older; and (3) individuals who accepted to participate in the survey. Sex and occupation were not restrictive criteria for enrollment. Exclusion criteria were (1) individuals with insufficient amount of serum for laboratory analysis and (2) individuals with incomplete epidemiological data. Selection of participants was performed by using a simple random sampling. In total, 282 subjects were included in this study; 94 of them were inhabitants of the San Dimas community located in a mountainous region; 82 were inhabitants of the Villa Montemorelos community located in a valleys region; and 106 were inhabitants of the Santa Clara community located in a semidesert region.

### 2.2. General Epidemiological Characteristics of Participants

A standardized questionnaire was used to obtain the sociodemographic and behavioral characteristics of the participants. Sociodemographic data including age, gender, birthplace, residence, educational level, socioeconomic status, and employment from participants was obtained. We determined the housing conditions of the participants by using Bronfman's criteria [14]. This tool assesses (1) crowding; (2) type of floors (ceramic, concrete, and soil); (3) availability of drinkable water (within the house, out of the house); and (4) form of elimination of excretes (flush toilet, latrine, or other). Furthermore, the educational level (years of education) of the head of the family was obtained. Behavioral data including consumption of unpasteurized milk or untreated water, consumption of unwashed raw vegetables or fruits, raising animals, traveling abroad, and contact with soil (gardening or agriculture) from participants was also obtained.

### 2.3. Laboratory Tests

Sera of the participants were analyzed for anti-*Leptospira* IgG antibodies by a commercially available enzyme immunoassay “*Leptospira* IgG ELISA test” kit (Diagnostic Automation Inc., Calabasas, CA). A serum sample was considered reactive for anti-*Leptospira* IgG antibodies when an absorbance reading equal to or higher than 0.5 optical density (OD) units was found. Weakly reactive samples were those with absorbance reading between 0.5 and 1.0 OD units. Strongly reactive samples or with high antibody levels were those with absorbance reading higher than 1.0 OD units. All immunoassays were performed following the instructions of the manufacturer. Positive and negative controls were analyzed in each run. According to the information provided in the kit's insert, the enzyme immunoassay used has a sensitivity of 100% and a specificity of 100%.

### 2.4. Statistical Analysis

We performed the statistical analysis with the aid of the software Epi Info version 7 and SPSS version 15.0. Sample size was calculated with the following parameters: a reference seroprevalence of 14.2% [15] as the expected frequency for the factor under study, 300000 as the population size from which the sample was selected, 5% confidence limits, and a 95% confidence level. The result of the sample size calculation was 187 subjects. We used the Pearson chi-squared test and the Fisher exact test (when values were less than 5) for initial comparison of frequencies among groups. Sociodemographic, housing, and behavioral variables with a *P* value ≤ 0.10 obtained in the bivariate analysis were included in a multivariate analysis to determine their association with* Leptospira* seropositivity. We calculated the odds ratios (OR) and 95% confidence intervals (CI) by using logistic regression analysis with the backward stepwise method. In addition, we used the Hosmer-Lemeshow goodness of fit test to evaluate the fitness of our regression model. A *P* value < 0.05 was considered statistically significant.

### 2.5. Ethical Aspects

Only residual serum samples and data from a previous survey [13] were used in the present study. The institutional ethical committee of the Mexican Social Security Institute in Durango City, Mexico, approved the previous study. The aims and procedures of the survey were explained to all participants and a written informed consent was obtained from all of them.

## 3. Results

Of the 282 rural subjects studied, 44 (15.6%) had anti-*Leptospira* IgG antibodies. Forty of them had low anti-*Leptospira* IgG antibody levels whereas four had high (>1.0 OD units) anti-*Leptospira* IgG antibody levels. A selection of sociodemographic and behavioral characteristics and housing conditions of participants and their correlation with* Leptospira* seropositivity is shown in Table 1. The mean age of rural subjects was 42.91 ± 17.53 years old (range 18–91 years). With respect to the sociodemographic, behavioral, and housing characteristics studied, the variables age, community of residence, trips to other Mexican states, and educational level of the head of the family had *P* values < 0.10 by bivariate analysis. Other sociodemographic, behavioral, and housing characteristics including gender, birthplace, residence, educational level, employment, socioeconomic status, raising animals, traveling abroad, consumption of unpasteurized milk or untreated water, consumption of unwashed raw vegetables or fruits, contact with soil, crowding, type of floors, availability of drinkable water at home, and form of elimination of excretes had *P* values > 0.10 by bivariate analysis. Multivariate analysis of sociodemographic, behavioral, and housing variables with *P* values < 0.10 obtained by bivariate analysis showed that* Leptospira* exposure was positively associated with a history of national trips (OR = 2.09; 95% CI: 1.05–4.16; *P* = 0.03) and poor (<3 years) education of the head of the family (OR = 2.96; 95% CI: 1.51–5.78; *P* = 0.001). The result of the Hosmer-Lemeshow test (*P* = 0.81) suggested a well-fitting of our regression model.

## 4. Discussion

The seroepidemiology of* Leptospira* infection in rural populations in northern Mexico is largely unknown. There are not currently reports on the magnitude of* Leptospira* exposure in humans in Durango, Mexico. Therefore, we performed this cross-sectional study to determine the seroprevalence and correlates of* Leptospira* exposure in general population in Durango State, Mexico. We found a 15.6% seroprevalence of anti-*Leptospira* IgG antibodies. Very few reports on the seroprevalence of* Leptospira* infection in Mexico exist. Therefore, we can hardly compare the seroprevalence of* Leptospira* exposure found in our study with others in Mexico. In a study in blood donors in Mexico City, researchers found a 7% seroprevalence of* Leptospira* infection by using microscopic agglutination assay [16]. The seroprevalence found in our study is higher than that reported in blood donors. Differences in sociodemographic characteristics and residence of the populations and in laboratory assays may explain the difference in seroprevalence among the studies. In a seroprevalence study of open population including rural and urban subjects in the southern Mexican state of Yucatan, a 14.2% seroprevalence was found. The seroprevalences of* Leptospira* exposure in Durango and Yucatan are comparable. However, this comparison should be taken with care since differences in population groups and laboratory assays exist among the studies. Researchers in Yucatan studied both rural and urban subjects whereas we studied only rural subjects. In addition, detection of anti-*Leptospira* antibodies in the study in Yucatan was performed by microscopic agglutination assay whereas we used an enzyme immunoassay. A serosurvey of* Leptospira* in the southern Mexican community of Jáltipan, Veracruz, reported a 4% seroprevalence of* Leptospira* exposure using an immunoassay [17]. The seroprevalence of* Leptospira* exposure found in our study is higher than that reported in Veracruz. It is not clear why the seroprevalence in the population in Durango was higher than the one in Veracruz. In both studies enzyme immunoassay was used; however, the commercial brands were different. Sensitivity and specificity of the enzyme immunoassay used in our study are 100% (according to the kit's insert) whereas the enzyme immunoassay used in the study in Veracruz had a sensitivity of 98.7% and a specificity of 94.2% [17]. In a study of rural population in the southern Mexican state of Chiapas, researchers found a 37.9% seroprevalence of* Leptospira* infection using the microscopic agglutination assay [18]. The seroprevalence of* Leptospira* exposure found in our study is lower than that reported in rural subjects in Chiapas. However, comparison of these seroprevalences should be cautious since differences in laboratory assays among the studies exist. We used an immunoassay whereas researchers in Chiapas used the microscopic agglutination assay [18]. Further research to compare enzyme immunoassay and microscopic agglutination assay for screening* Leptospira* exposure is needed. The higher seroprevalence in rural population in Chiapas than that found in rural Durango might be due to differences in environment between both Mexican states. Flooding was associated with* Leptospira* exposure in Chiapas [18], whereas this environmental condition is rarely observed in Durango.

In the present study,* Leptospira* exposure was associated with a history of national trips and poor (<3 years) education of the head of the family. To the best of our knowledge, this is the first report of an association of* Leptospira* exposure with a history of national trips and poor education of the head of the family in a cross-sectional study. Very little information about* Leptospira* infection and traveling exists. A leptospirosis case in Germany occurred in a man after a 4-week trip to the north of Thailand [19]. An outbreak of leptospirosis in persons who had returned from a white-water rafting trip in Costa Rica was reported [20]. In another report, a Dutch male developed leptospirosis when returning from a 5-month trip to China [21]. Similarly, a Japanese man developed leptospirosis after a trip to Bali Islands, Indonesia [22]. In the USA, a case of leptospirosis in a girl who had prolonged* Leptospira* urinary shedding following a trip to Costa Rica [23] and a severe case of leptospirosis in a man who had just returned from a 2-month trip to Southeast Asia [24] were reported. In the present study, the finding that* Leptospira* exposure was associated with national but not with international trips might suggest that* Leptospira* exposure occurred domestically in Mexico. On the other hand, it is unclear why* Leptospira* exposure was associated with low education of the head of the family. There are no reports of this association in the medical literature. However, the association of* Leptospira* exposure with low education of the head of the family might reflect the following: (1) no optimal education to the family members concerning hygiene practices to avoid infections; (2) poor environment conditions including poor sanitation and contact with contaminated water or infected animals that may favor transmission of* Leptospira*; and (3) other unknown reasons. Low education was an import risk factor for* Leptospira* infection among butchers and slaughterhouse workers in Jamaica [25]. Further research to elucidate the role of low education of the head of the family on* Leptospira* exposure is needed. Contact with animals is a well-known factor associated with* Leptospira* infection [7]; however, this factor was not associated with seropositivity to* Leptospira* in our study.

## 5. Conclusions

We demonstrated serological evidence of* Leptospira* exposure in rural population in the northern Mexican state of Durango, Mexico. The contributing factors associated with* Leptospira* exposure found in the present study may be useful for optimal planning of preventive measures against* Leptospira* infection. Further research on the epidemiology of* Leptospira* infection in Mexico is needed.

## Figures and Tables

**Table 1:** Bivariate analysis of a selection of sociodemographic, behavioral, and housing variables and seroprevalence of *Leptospira* exposure.

Characteristics	Number of subjects tested	Positive ELISA results		*P* value
		Number	%	
Gender				
Male	62	10	16.1	0.89
Female	220	34	15.5	
Age groups (years)				
30 or less	75	13	17.3	0.06
31–50	120	12	10.0	
>50	87	19	21.8	
Community				
San Dimas	94	9	9.6	0.01
Villa Montemorelos	82	21	25.6	
Santa Clara	106	14	13.2	
Educational level				
No education	27	4	14.8	1.00
Education	255	40	15.7	
Occupation				
Employed^a^	66	11	16.9	0.78
Unemployed^b^	216	33	15.3	
Socioeconomic status				
Low	218	37	17.0	0.24
Medium	64	7	10.9	
Raising farm animals				
Yes	234	39	16.7	0.27
No	48	5	10.4	
Dogs at home				
Yes	37	8	21.6	0.27
No	245	36	14.7	
Traveled abroad				
Yes	44	9	20.5	0.33
No	238	35	14.7	
National trips				
Yes	148	29	19.6	0.05
No	134	15	11.2	
Soil contact				
Yes	250	40	16	0.79
No	32	4	12.5	
Source of drinking water				
Home	172	31	18	0.16
Out of home	110	13	11.8	
Sewage disposal				
Pipes	130	19	14.6	0.67
Latrine, other	152	25	16.4	
Crowding at home				
No	62	13	21	0.18
Yes	220	31	14.1	
Education of the head of family				
Up to 3 years	113	27	23.9	0.002
More than 3 years	169	17	10.1	
Floor at home				
Ceramic	19	2	10.5	0.19
Concrete	184	34	18.5	
Soil	79	8	10.1	

^a^Employed: agriculture, construction worker, factory worker, business, professional, and other.

^b^Unemployed: housekeeping, students, or no occupation.
